# Management of a respiratory syncytial virus outbreak in a memory care unit at a long-term care facility

**DOI:** 10.1017/ash.2025.10048

**Published:** 2025-07-11

**Authors:** Alaina S. Ritter, Debbie Manderville, Laura Netardus, Amy Y. Vittor

**Affiliations:** 1 Division of Infectious Diseases, Malcom Randall Veterans Affairs Medical Center, Gainesville, FL, USA; 2 Division of Infectious Diseases and Global Medicine, University of Florida College of Medicine, Gainesville, FL, USA; 3 Infection Control Division, Malcom Randall Veterans Affairs Medical Center, Gainesville, FL, USA

## Abstract

**Background:**

Respiratory syncytial virus (RSV), although frequently reported in pediatric populations, is also associated with significant morbidity and mortality in vulnerable adults. From an Infection Control perspective, it is therefore of particular concern in hospital and long-term care settings.

**Objective:**

We report an RSV outbreak that occurred in the memory care unit of a Veterans Affairs-affiliated long-term care facility where the characteristics of the resident population posed unique challenges to halting transmission.

**Setting:**

The outbreak occurred in a 30-bed unit within a 230-bed Veterans Affairs-affiliated long-term care facility in Florida.

**Methods:**

An investigation was performed in coordination with the local Department of Health. All residents on the affected unit had dementia with resulting difficulty participating in infection prevention measures, including isolation, masking, and hand hygiene. Interventions implemented included twice weekly RSV testing, enhanced cleaning protocols, staggered mealtimes/outdoor dining, and cancellation of group activities, visitations, and new admissions. A retrospective case–control study was performed to assess for potential risk factors for acquiring RSV.

**Results:**

Over a 21-day period in 2022, 20 out of 29 residents tested positive for RSV within the affected unit. No other units were involved. Univariate analysis did not find any statistically significant risk factors for acquiring RSV infection, although small sample size may have impacted the results.

**Conclusions:**

A multifaceted approach was ultimately successful in preventing ongoing transmission of RSV within and beyond the unit. The infection control techniques utilized in this unique patient population could potentially be applicable to challenging outbreak situations at other facilities.

## Introduction

Respiratory syncytial virus (RSV) is a significant public health concern, especially among vulnerable patient populations in hospital and long-term care facility (LTCF) settings.^1^ Affected patients are at increased risk of morbidity, mortality, and extended hospital stays.^2,3^ RSV, a member of the Paramyxoviridae family, is generally transmitted through large droplets from nasopharyngeal secretions.^4–6^ The incubation period of the virus is typically two to eight days and patients are contagious one to two days before symptom onset.^7^ Patients may remain asymptomatic or may develop symptoms including cough, nasal congestion, sore throat, fever, and dyspnea. In severe cases, patients may develop respiratory failure, which can result in death.^3,8^ Treatment is typically supportive.^9^

Although RSV is often reported in pediatric populations, it is also of clinical importance in adults, especially those in communal settings or LTCF’s.^3,8,10^ RSV infection complications result in the death of approximately 10,000 to 11,000 elderly patients annually in the USA.^11,12^ RSV was initially recognized as a public health concern in LTCF’s in the 1970s.^13,14^ Outbreaks in congregate settings can result in high attack rates of up to 85% with up to 30% of patients developing pneumonia and a death rate of approximately 5%.^10,15–17^

Risk factors for developing severe disease/requiring hospitalization include age greater than 65 years old, immunocompromise, and multiple comorbidities including cardiopulmonary disease.^3,8,18^ Underlying conditions such as asthma, chronic obstructive pulmonary disease (COPD), and congestive heart failure (CHF) are often associated with hospitalizations related to RSV.^2,3,19^ RSV infection results in incomplete immunity and patients may develop repeat infections during their lifetime.^20^

Infection control measures are critical for preventing transmission in outbreak settings, especially given that until 2023, there was no approved vaccine to prevent RSV in adults.^21^ Interventions such as the use of personal protective equipment (PPE; such as gowns, gloves, masks, and eye protection), hand hygiene, isolation of affected patients, cleaning of contaminated surfaces, and cohort nursing have been reported as effective in preventing and controlling nosocomial outbreaks.^1,22^ We describe an RSV outbreak in a Veterans Affairs-affiliated LTCF that occurred in the spring of 2022, with the goal of identifying successful strategies that could apply to similar scenarios at other facilities.

## Materials and methods

This study was approved by our institutional research compliance office. The need for patient consent was waived given that the study was determined not to meet criteria for human patients research through use of the VA Electronic Determination Aid (VAEDA). All patients were treated according to clinical judgment and infection control practices in order to control the outbreak according to local guidelines.

The LCTF that experienced the RSV outbreak is a 230-bed facility consisting of four separate units. Only one unit, a memory care unit with 30 single-bed rooms, was affected. All cases were identified over a 21-day period in the spring of 2022. As part of the outbreak investigation, discussions were held with facility leadership and staff members to gather additional pertinent information. All of the residents in the affected unit suffered from cognitive impairment/dementia and wandered freely within the unit. In general, residents had little understanding or comprehension of hand hygiene or infection prevention measures. No RSV vaccine was available at the time of this outbreak.

Several staff members had suffered from respiratory symptoms around the time the first residents became ill. One staff member subsequently self-reported a positive test for RSV done at an outside location. This staff member had worked on day 11 of the outbreak but called out sick the following day. Due to staffing shortages, staff members were also covering from other units. Each staff member typically interacted with many of the residents daily and served meals in the communal dining room. In addition, group activities such as chaplain visits or physical therapy sessions were often held in communal areas at the time the outbreak started. Outside visitations were limited due to the concurrent COVID-19 pandemic, and no COVID-19 cases were identified during the course of this RSV outbreak.

In order to address the outbreak, Hospital Epidemiology and Infection Control collaborated with the local Department of Health to implement a number of infection control measures. Prior to the outbreak, all residents were screened twice weekly for COVID-19. After the first RSV-positive resident was identified, residents with symptoms concerning for RSV were additionally screened with a multiplexed real-time RT-PCR test (Xpert^®^ Xpress Flu/RSV test; Cepheid^®^, Sunnyvale, CA). In addition, twice weekly influenza/RSV testing of all residents without a recent RSV diagnosis was started on day 13 of the outbreak once approval from hospital leadership and coordination with the microbiology lab were finalized. Use of scheduled testing ensured that asymptomatic or mildly symptomatic residents with RSV could be more expediently identified.

Residents with RSV were placed on contact precautions (gown/gloves) and encouraged to stay in their rooms. Isolation precautions were maintained for 10 days after diagnosis and were subsequently discontinued if respiratory symptoms had resolved. Due to the ongoing COVID-19 pandemic, universal masking was required of all staff and residents. Staff members reported excellent compliance with masking, which was monitored and enforced by unit supervisors, although resident compliance was extremely difficult to enforce due to cognitive impairment. RSV-positive residents were served meals in their rooms when possible or outdoors on the patio, and for those who were not agreeable, efforts were made to serve RSV-negative and positive residents at separate times in the dining room with cleaning of the high-touch surfaces in between the two groups. Nursing staff in the unit made additional efforts to clean high-touch surfaces with disinfectant wipes on a regular basis, and environmental services was asked to increase the frequency of cleanings during the week and on weekends. All group activities were cancelled and the unit remained closed to visitors. Admissions to the unit and transfers to other units were halted during the outbreak to avoid bringing new cases of RSV into the unit and prevent spread to other units. Formal multidisciplinary meetings were held on day 13 and day 16 of the outbreak so that Hospital Epidemiology and Infection Control could review and reinforce the above measures with facility staff and discuss challenges to implementation.

Staff members with respiratory symptoms were asked not to come to work and efforts were made to prevent staff working on the affected unit from floating to other units not affected by the outbreak. RSV testing of asymptomatic staff members was not feasible. There was heightened awareness of symptom monitoring and testing in the entire facility during and after the outbreak, and twice weekly testing in the affected unit was maintained for two weeks after the end of the outbreak.

At the conclusion of the outbreak, we performed a retrospective case–control study to identify potential risk factors for acquiring RSV.

### Statistical analysis

Due to the small sample size, only descriptive data is shown. Data were analyzed using STATA v14.2 (Statacorp, College Station, TX) and RStudio (RStudio, PBC, Boston, MA). Group means were compared using the Student t-test and binary results were compared using the chi-squared test.

## Results

**Figure 1.**
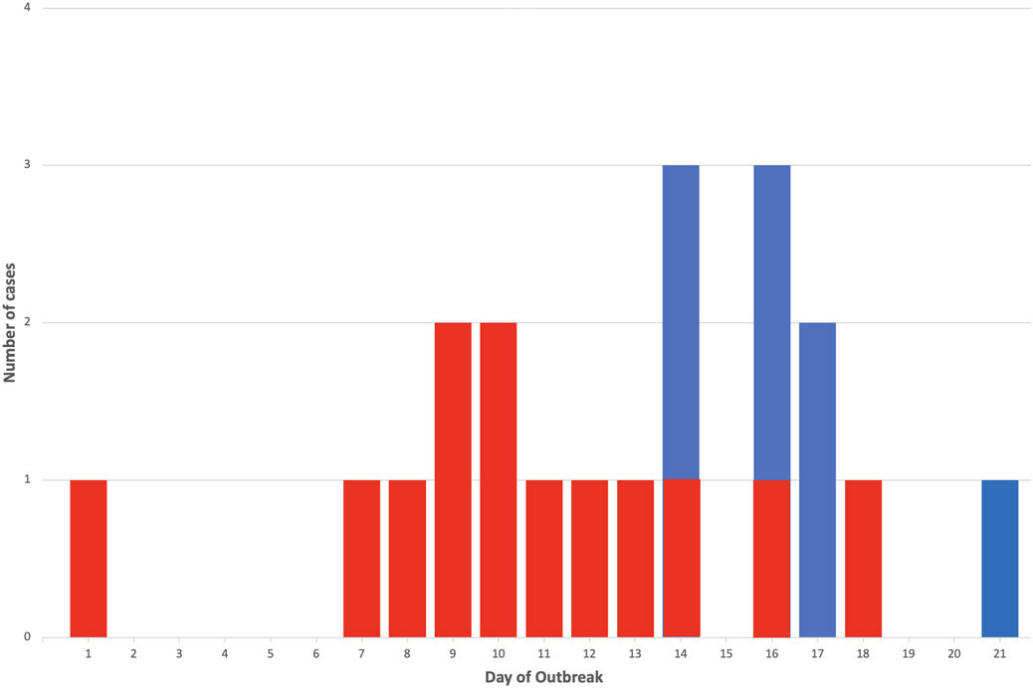
Epidemiological curve of the RSV outbreak in 2022. Symptomatic cases are depicted in red, and asymptomatic cases are in blue.

**Figure 2.**
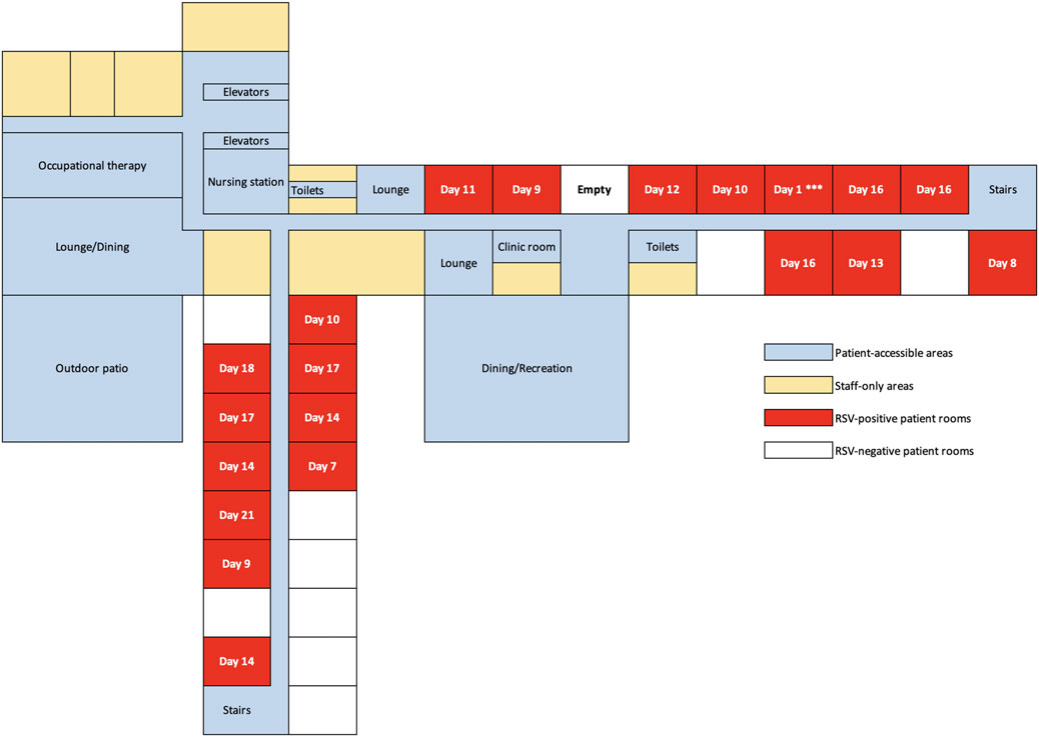
Floor plan layout of the affected LCTF unit. The day each positive RSV test occurred is listed. Day 1 *** is the index case.

During the course of the 21-day outbreak, a total of 20 residents tested positive for RSV out of a total of 29 residents on the unit. The last case of RSV was detected on day 21, signaling the end of the outbreak. See Figure 1 for the distribution of RSV cases by day and Figure 2 for the physical layout of the affected unit and documentation of timing of infection by room. All rooms were single-bed and had a private bathroom. All of the residents on the unit were male. Of the residents who were affected, eight were asymptomatic and the other 12 had at least one associated respiratory symptom. During the course of the outbreak, two residents required hospitalization. The first resident was hospitalized on day 13 of the outbreak for six days after developing fevers, cough, dyspnea, and malaise; he tested positive for RSV on admission and was found to have pneumonia. The second resident suffered a fall and was hospitalized on day 18 of the outbreak for four days; he tested positive for RSV on admission and was noted to have malaise, wheezing, and hypoxia. One mildly symptomatic resident diagnosed with RSV on day 16 suffered a fatal acute stroke 23 days later. This resident had a history of a prior stroke and CHF in addition to other comorbidities, but it was unclear if his recent RSV infection may have been a contributing factor.

Patient characteristics and the presence of comorbidities are shown in Table 1. There were no significant differences between the RSV-infected and uninfected groups. Similarly, there were no significant differences between the symptomatic and asymptomatic groups (data not shown).

**Table 1.** Characteristics of the RSV Cohort. No statistically significant differences were observed between the RSV positive and negative groups

Characteristics	RSV positive, n = 20 (95% CI)	RSV negative, n = 9 (95% CI)
Age (years)	76.7 (73.0, 80.3)	74.2 (66.4, 82.0)
BMI (kg/m2)	27.5 (24.5, 30.6)	25.1 (21.2, 29.0)
Race/ethnicity (% White)	75% (50.3, 89.9)	88.9% (44.4, 98.8)
% Prior smoker	55% (31.5, 76.9)	66.7% (29.9, 92.5)
% with diabetes	40% (19.1, 63.9)	55.6% (21.2, 86.3)
% with hypertension	80% (56.3, 94.3)	77.8% (40, 97.2)
% with hyperlipidemia	75% (50.9, 91.3)	66.7% (30, 92.5)
% with Chronic Kidney Disease	20% (5.7, 34.7)	0% (0, 33.6)
% with Cerebrovascular Accident	40% (19.1, 63.9)	22.2% (2.8, 60)
% with Congestive Heart Failure	20% (5.7, 43.7)	11.1% (0.3, 48.2)
% with Coronary Artery Disease	50% (27.2, 72.8)	33.3% (7.5, 70.1)
% with Chronic Obstructive Pulmonary Disease	20% (5.7, 43.7)	44.4% (13.7, 78.8)
% with Obstructive Sleep Apnea	10% (1.2, 31.7)	22.2% (2.8, 60)
% with Malignancy	35% (15.4, 59.2)	22.2% (2.8, 60)

## Discussion

We investigated an RSV outbreak in one unit of an LCTF that affected 20 out of 29 residents over 21 days in the spring of 2022. No concurrent or subsequent cases of RSV were detected in any other units, demonstrating the successful prevention of spread beyond the affected unit. Of the 20 RSV-positive residents, 12 had at least one associated respiratory symptom, two required hospitalization, and another resident died after suffering an acute stroke of uncertain etiology 23 days after his RSV diagnosis.

RSV transmission during this outbreak likely occurred between residents as well as staff members who worked on the unit just prior to developing respiratory symptoms. Implementation of best practices in infection control including isolation, hand hygiene, and cohort nursing was especially difficult given the unique behavioral challenges posed by the residents in our memory care unit. Residents frequently interacted with other residents and staff and could not be redirected. Encouraging hand hygiene was also challenging given cognitive impairment. In addition, although all rooms on the unit were private, we were unable to remove RSV-negative residents from the unit given the risk of spreading infection to other units as well as resident challenges adapting to a new environment.

RSV is a significant cause of morbidity, mortality, and healthcare expenditures especially in LTCF residents.^23^ A study by Barrett et al, for example, reported an RSV outbreak involving 20 veterans across two units in a Veterans Affairs-affiliated LTCF. This outbreak was managed by implementing strict infection control measures including using rapid influenza/RSV tests to identify cases early, increasing cleaning measures, emphasizing hand hygiene, ensuring RSV-positive residents were in single rooms or cohorted, cancelling group activities, and closing the unit to new admissions.^7^

We managed our institutional outbreak through a multi-faceted approach. Holding multidisciplinary meetings between Hospital Epidemiology, Infection Control, and facility staff was helpful to strategize and reinforce interventions. We remained in close communication with the local Department of Health regarding the evolving outbreak. In contrast to the approach in Barrett et al., we were able to prevent outside visitation and use staggered dining times, and we were also uniquely able to provide outdoor dining on the patio given the LCTF layout.^7^ We were also able to implement twice weekly testing for all residents without a recent RSV diagnosis, whereas testing of asymptomatic patients was instead done on a case-by-case basis in this prior study.^7^ While testing asymptomatic staff members for RSV might have detected staff with an early or mild infection and thus minimized their contact with residents, this was not feasible for us. We were able to halt new admissions/transfers and group activities, and we reinforced the use of appropriate PPE and increased the frequency of cleaning in the unit with a special emphasis on high-touch surfaces. We also avoided having staff from the affected unit float to other units within the facility. As a result of the above interventions, we were able to halt the outbreak and prevent spread to neighboring units despite the behavioral challenges posed by residents on this unit.

Limitations of our study include difficulty confirming RSV infection in facility staff, as symptoms of illness were self-reported and testing of asymptomatic staff for RSV was not feasible during the course of the outbreak. In addition, since RSV infection does provide incomplete immunity, it is possible that some of the unit residents did not contract the virus due to existing protective immunity. The small patient population size in our study may have also contributed to the inability to detect significant risk factors on univariate analysis.

In conclusion, RSV outbreaks are of significant concern especially in LCTF’s and there should be a low threshold to test for RSV in symptomatic and at-risk residents in these facilities. The RSV outbreak we describe occurred in a setting where the affected residents suffered from dementia/cognitive impairment, making standard infection control techniques less effective. A multidisciplinary collaborative approach and the implementation of interventions targeted towards efficacy in this LTCF population, as discussed in our manuscript, ultimately proved effective in halting the outbreak. The unique combination of interventions described in this report could potentially be applicable to other facilities facing similar challenges in preventing the spread of RSV or other respiratory infections.
